# A taxonomic note on the helicoid land snail genus *Traumatophora* (Eupulmonata, Camaenidae)

**DOI:** 10.3897/zookeys.835.32697

**Published:** 2019-04-05

**Authors:** Min Wu

**Affiliations:** 1 School of Life Sciences, Nanjing University, Xianlindadao 163, Qixia, Nanjing 210023, China Nanjing University Nanjing China

**Keywords:** Bradybaeninae, Camaeninae, China, distribution, *
Traumatophora
*, Zhejiang

## Abstract

*Traumatophoratriscalpta* (Martens, 1875) is reported for the first time from the Tianmushan Mountains, Zhejiang Province, and its morpho-anatomy is described based on this new material. The genus *Traumatophora* is redefined on the basis of both shell and genital anatomy of its type species. The presence of the dart apparatus suggests this genus belongs to the subfamily Bradybaeninae rather than to the Camaeninae. This genus is distinguished from all other Chinese bradybaenine genera by the combination of the following key morphological characteristics: embryonic shell smooth, palatal teeth present, dart sac tiny with rounded proximal accessory sac that opens into a dart sac chamber, mucous glands well developed, entering an accessory sac through a papilla, epiphallic papilla absent, flagellum present. A comparison is also presented of Chinese bradybaenine genera with known terminal genitalia.

## Abstract (cn)

摘 要 本文首次从浙江省天目山报道了三痕创螺 *Traumatophoratriscalpta* (Martens, 1875)。基于贝壳和生殖系统解剖，对创螺属*Traumatophora*进行了修订。恋矢器官在创螺属中出现，表明创螺属归属于巴蜗牛亚科而非坚螺亚科。创螺属以其鉴别特征“胚螺层光滑。壳口腭具齿。矢囊极小。具极发达的开口于矢囊腔的球状基副矢囊 (proximal accessory sac)。粘液腺亦极发达；通过粘液腺乳突进入副矢囊。成荚器乳突阙如。具鞭状体”，可很好地区别于其他所有巴蜗牛亚科中生殖系统端部解剖结构已知的属。本文亦对已知生殖系统端部解剖结构的中国巴蜗牛亚科各类群进行了比较。

## Introduction

Camaenidae Pilsbry, 1895 (Bouchet et al. 2017) is a helicoid land snail family mainly distributed in Asia, Australia and some Pacific islands (Solem 1992, Schileyko 2003). In China, members of the Camaeninae (i.e., taxa lacking stimulatory organs; Wade et al. 2006, 2007; Hirano et al. 2014) are distributed south of the Qingling Mountains on the mainland, and on Taiwan and Hainan Island. They are grouped into eleven genera, namely *Camaena* Albers, 1850, *Amphidromus* Albers, 1850, *Ganesella* Blanford, 1863, *Satsuma* A. Adams, 1868, *Stegodera* Martens, 1876, *Traumatophora* Ancey, 1887, *Trichelix* Ancey, 1887, *Moellendorffia* Ancey, 1887, *Trichochloritis* Pilsbry, 1891, *Camaenella* Pilsbry, 1893 and *Moellendorffiella* Pilsbry, 1905 (Pilsbry 1890, 1894; Yen 1939; Chang 1989; Schileyko 2003; Wu et al. 2008; Du et al. 2013). Among them, only the genera *Camaena*, *Camaenella*, *Moellendorffia*, *Trichelix* and *Satsuma* have been defined by genital features (Chang 1989, Solem 1992, Azuma 1995, Schileyko 2003, Wu et al. 2008). In comparison to the genera in the Bradybaeninae which have a dart apparatus as a rule, the dart apparatus-absent camaenine genera of China share the following characteristics: dart sac absent; both an epiphallic papilla and flagellum present; and penial retractor muscle inserting on epiphallus.

In this paper, the genus *Traumatophora*, previously assigned to the Camaenidae (= Camaeninae sensu Bouchet et al. 2017), is reported from Zhejiang for the first time. Based on shell morphology, the genus *Traumatophora* is thought by some authors to be near the genus *Stegodera* (Ancey 1887, Schmacker and Boettger 1894). The genital anatomy, newly revealed in this work, suggests that this genus rather belongs to the subfamily Bradybaeninae.

## Materials and methods

A living specimen was relaxed by drowning in water before being transferred to 70% ethanol for fixation, which was replaced with ethanol of the same concentration after three days. The shell and genitalia were measured with digital vernier callipers (genitalia from photo) to the nearest 0.1 mm. Whorl number was recorded as described by Kerney and Cameron (1979), with 0.125 whorl accuracy. Soft parts were measured after the specimens were sufficiently fixed in 70% ethanol. Directions used in descriptions: proximal = towards the genital atrium; distal = away from the genital atrium.

Abbreviations: AS – accessory sac; At – atrium; BC – bursa copulatrix; BCD – bursa copulatrix duct; DS – dart sac; DVM – membranous sac surrounding terminal genitalia; Ep – epiphallus; Fl – flagellum; FO – free oviduct; HBUMM – mollusc collection of the Museum of Hebei University, Baoding, China; MG – mucous glands/number of mucous gland duct; MGP – papilla distally leading to mucous glands on inner wall of accessory sac; P – penis; PAS – proximal accessory sac, a blind sac on proximal dart sac and opening into dart sac chamber or not; PLs – poly-layered structure in dart sac and/or accessory sac, produced by wavy and spongy connective tissue; PR – penial retractor muscle; PS – penis sheath; Va – vagina; VD – vas deferens; ZMB/Moll – Museum für Naturkunde, Berlin-Malakologie.

## Systematics

### Helicoidea Rafinesque, 1815

#### Camaenidae Pilsbry, 1895

##### Bradybaeninae Pilsbry, 1898

###### Traumatophora

Taxon classificationAnimaliaStylommatophoraCamaenidae

Ancey, 1887

Traumatophora Ancey, 1887: 54; Pilsbry 1890: 6; Pilsbry 1894: 145–146, subgenus of Plectopylis Benson, 1860; Schmacker and Boettger 1894: 173, subgenus of Stegodera Martens, 1876; Yen 1939: 125; Schileyko 2003: 1512–1513.

####### Type species.

*Helixtriscalpta* Martens, 1875; original designation.

####### Diagnosis.

Embryonic shell smooth. Palatal teeth present. Dart sac tiny. A ball-shaped proximal accessory sac with opening leading to dart sac chamber. Mucous glands numerous; developed; entering accessory sac by a papilla. Epiphallic papilla absent. Flagellum present.

####### Description.

Shell depressed; solid; with approximately five moderately convex whorls. Last whorl descending behind aperture. Embryonic whorls smooth. Aperture oblique; with three palatal lamellar teeth. Outer surface of body whorl with longitudinal depressions corresponding to teeth. Aperture margins reflexed. Umbilicus moderately broad (Schileyko 2003, slightly altered).

Membranous sac surrounding terminal genitalia absent. Penis sheath absent. Epiphallic papilla wanting. Penial caecum absent. Flagellum present. Dart sac tiny. Accessory sac developed; large; with transversal sphincter muscles. Mucous glands numerous; extremely developed; entering accessory sac by a papilla. A ball-shaped proximal accessory sac with opening leading to dart sac chamber. Poly-layered structure in dart apparatus absent (this study).

####### Distribution.

S China (extant range: Jiangxi, Hubei, Fujian and Zhejiang; Pleistocene: Jiangsu).

####### Remarks.

The genus *Traumatophora* is transferred herein to the subfamily Bradybaeninae based on the presence of a dart apparatus that is structurally similar to that of other genera in this subfamily. Features typical of the genus include presence of a penis sheath, flagellum, accessory and proximal accessory sac, mucous gland papilla and the absence of an epiphallic papilla, penial caecum, poly-layered structure, and a membranous sac surrounding the terminal genitalia; on this basis the genus *Traumatophora* is well distinguished from all the other anatomically known Chinese bradybaenine genera [Table 1; note: at transition of the penis-epiphallus in *Acusta* (Wu 2004: fig. 18E), *Laeocathaica* (Wu 2004: fig. B), *Eueuhadra* (Wu 2004: figs 7B, 7E) and *Aegistohadra* (Wu 2004: fig. 6C), the structures once called an “epiphallic papilla” are too invisibly tiny (especially compared with those in *Pfeifferia* Gray, 1853, *Pliocathaica* Andreae, 1900, etc.) to a true epiphallic papilla. Anatomy information of *Armandiella* Ancey, 1883 comes from *A.sarelii* (Martens, 1867) (HBUMM01113 specimen-3, collection information lost)]. However, until now detailed information on the anatomy of the terminal genitalia of the following genera Chinese endemic genera still remains unknown: *Campylocathaica* Andreae, 1900 and *Xerocathaica* Andreae, 1900.

###### Traumatophora triscalpta

Taxon classificationAnimaliaStylommatophoraCamaenidae

(Martens, 1875)

**Figure 1.**
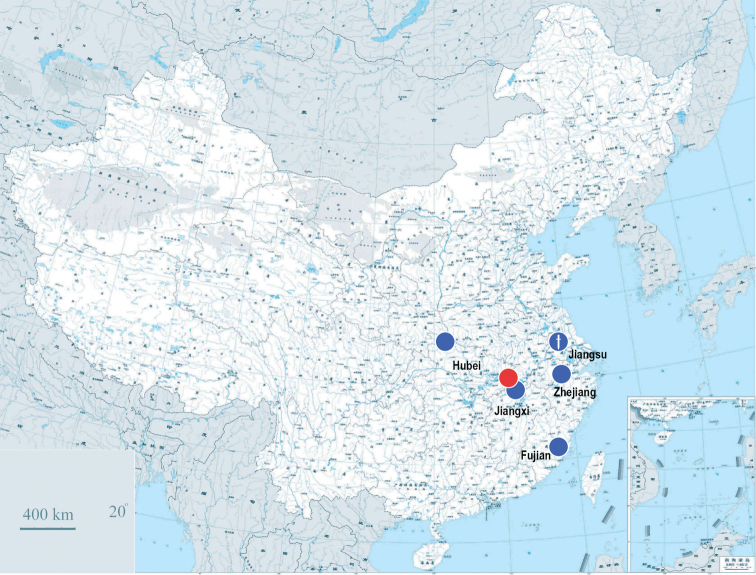
Known occurrence records of *Traumatophora* spp. Blue dots: *Traumatophoratriscalpta* (Martens, 1875), fossil locality with sword mark; red dot: *T.fraterminor* (Gredler, 1884).

**Figure 2.**
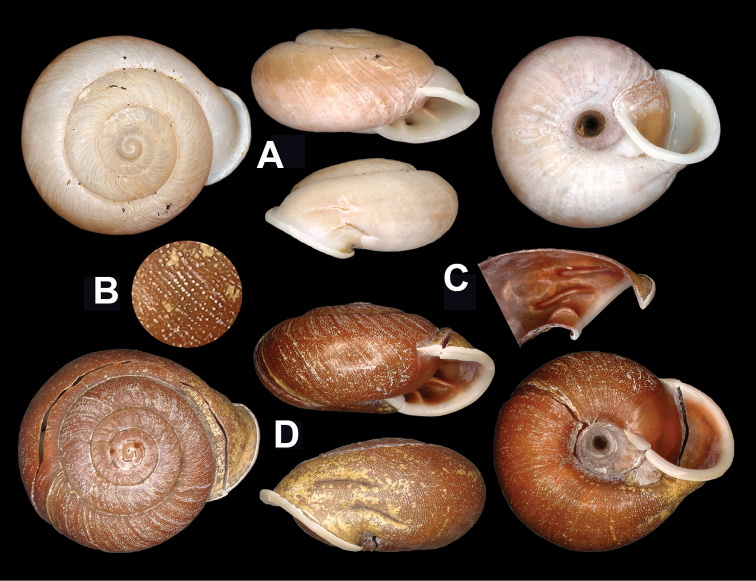
*Traumatophoratriscalpta* (Martens, 1875). **A** HBUMM08236-specimen 1, shell **B** magnified shell surface, magnified showing growth lines breaking into granules **C** palatal part of body whorl, showing lamellar teeth **D** shell **B–D** HBUMM06875 specimen-2.

**Figure 3.**
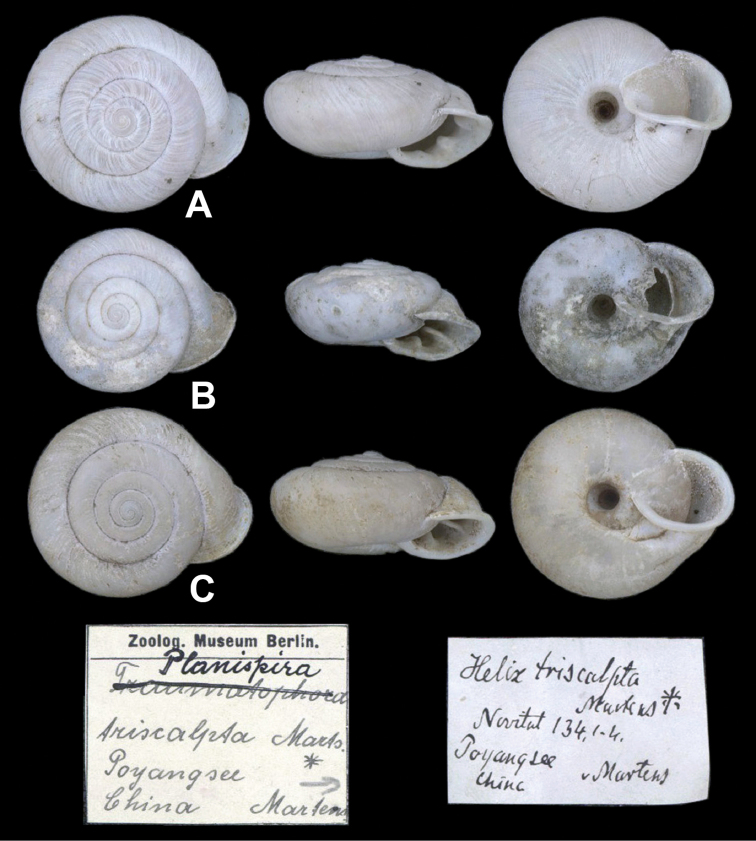
*Helixtriscalpta* von Martens, 1875 **A–C** syntypes, ZMB/Moll-109875; China, Province Kiangsi: Poyang-Yu (Lake Poyang); 3 dried shells; leg.: von Martens. The largest shell has a major diameter 31.0 mm.

**Figure 4.**
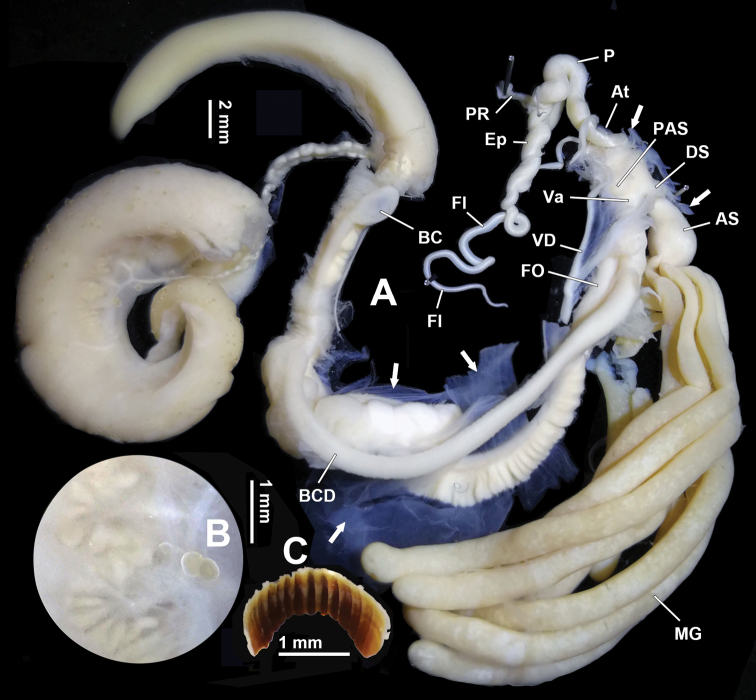
*Traumatophoratriscalpta* (Martens, 1875). HBUMM06875 specimen-2. **A, B** genitalia **A** general view, the remnant membrane after dissection is arrowed **B** magnified hermaphroditic gland **C** jaw. AS–accessory sac; At–atrium; BC–bursa copulatrix; BCD–bursa copulatrix duct; DS–dart sac; Ep–epiphallus; Fl–flagellum; FO–free oviduct; MG–mucous glands; P–penis; PAS–proximal accessory sac, a blind sac on proximal dart sac opening into dart sac chamber or not; PR–penial retractor muscle; Va–vagina; VD–vas deferens.

**Figure 5.**
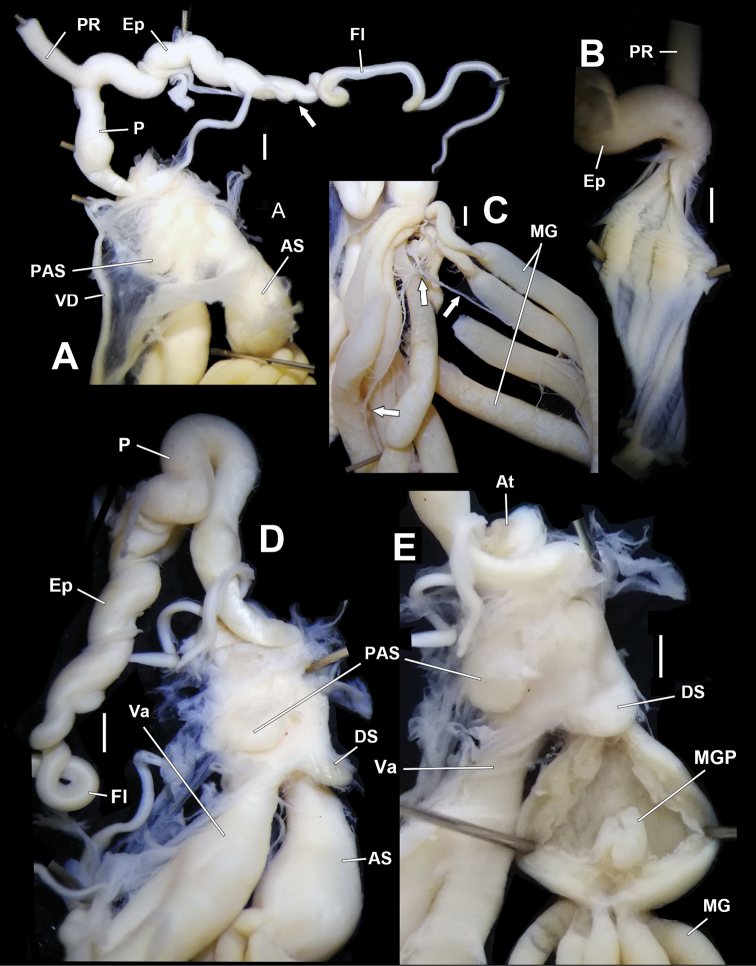
*Traumatophoratriscalpta* (Martens, 1875). Genitalia, HBUMM06875 specimen-2. **A** male part, vas deferens insertion arrowed **B** exposed penis **C** mucous glands, showing nerve fibres (arrowed) connecting mucous gland ducts **D** dart apparatus, peculiarly showing the dart sac and the ball-shaped caecum **E** exposed accessory sac, showing mucous gland entering papilla Scale bars: 1 mm. AS–accessory sac; At–atrium; DS–dart sac; Ep–epiphallus; Fl–flagellum; MG–mucous glands; MGP– papilla distally leading to mucous glands on inner wall of accessory sac; P–penis; PAS–proximal accessory sac, a blind sac on proximal dart sac opening into dart sac chamber or not; PR–penial retractor muscle; Va–vagina; VD–vas deferens.

**Figure 6.**
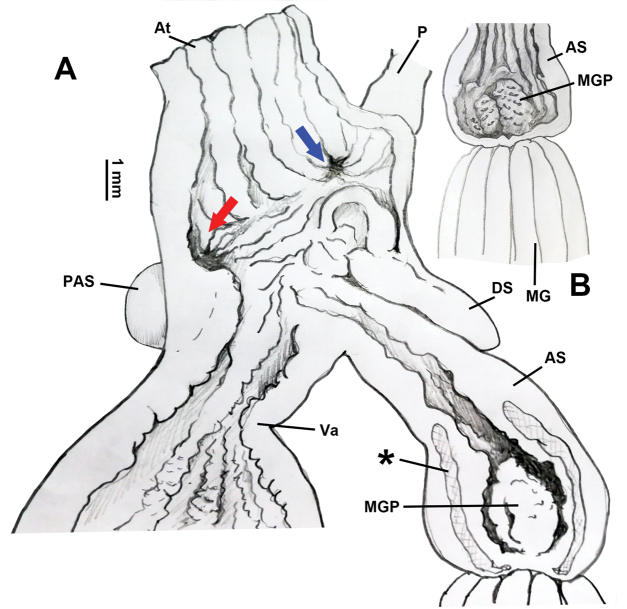
*Traumatophoratriscalpta* (Martens, 1875). Terminal genitalia, HBUMM06875 specimen-2. **A** exposed terminal genitalia. Blue arrow, indicating penis entrance; red arrow, indicating PAS opening leading to dart sac chamber **B** exposed accessory. AS–accessory sac; At–atrium; DS–dart sac; MG–mucous glands; MGP–papilla distally leading to mucous glands on inner wall of accessory sac; P–penis; PAS–proximal accessory sac, a blind sac on proximal dart sac opening into dart sac chamber or not; Va–vagina; *–sphincter muscles.

Figs 1 , 2 , 3 , 4 , 5 , 6

Helix triscalpta Martens, 1875a: 2; Martens 1875b: 185–186; Heude 1882: 35–36, pl. 15, figs 7, 7a, 7b; Gredler 1884: 137; Möllendorff 1884: 388.
Helix (Traumatophora) triscalpta Pilsbry 1890: 6, 8, pl. 1, figs 1–8.
Stegodera (Traumatophora) triscalpta Schmacker and Boettger 1894: 173.
Traumatophora triscalpta Yen 1939: 126, pl. 13, fig. 7; Yen 1942: 271–272; Yen 1943: 297; Schileyko 2003: 1512, fig. 1949.

####### Material examined.

*Helixtriscalpta* von Martens, 1875, syntypes, ZMB/Moll-109875; Poyang-Yu (Lake Poyang), Kiangsi Province, China; 3 dried shells (major diameter of three shells: 31.0 mm, 30.5 mm, 26.3 mm. Measurement made by Christine Zorn); leg. von Martens. Tianmushan, Zhejiang Province, China; 1 fully matured empty shell (HBUMM06875 specimen-1) and 1 full matured animal (HBUMM06875 specimen-2, body whorl was partially removed to take out whole soft parts), May, 2016; coll. Zhou, Dakang (Beijing Botanical Garden). A piece of foot of HBUMM06875 specimen-2 was cut off and preserved in 99.7% alcohol at –20 °C.

####### Description.

Shell (Figs 2, 3). Clearly depressed; thick and solid; dextral. Whorls convex. Suture impressed. Umbilicus approximately one fifth of shell major diameter. Bottom-umbilicus transition changed gently. Columella oblique. Columellar lip only slightly covering umbilicus. Protoconch smooth. On teleconch spiral furrows absent. Aperture strongly oblique; not sinuate at peristome. Body whorl descending abruptly in front. Shell surface without ribs. Growth lines fine and broken into granules which are distributed evenly on whorls except on protoconch (Fig. 2B); not accompanied by irregular thickenings. Shell not perforated. Adult shell not hairy or scaly. Adult body whorl rounded at periphery; with bottom convex. Ring-like thickening within aperture absent. Aperture with three baso-palatal lamellar teeth (Figs 2C, 3A–C). Palatal tooth near columella shortest, approximately half length of other two teeth. Outer surface of body whorl with longitudinal depressions corresponding to teeth (Figs 2A, D, 3A–C). Peristome thin; white; narrowly and uniformly reflexed. Callus indistinct. Shell dull to somewhat glossy; uniformly in reddish brown; bandless. Measurements (HBUMM06875, n = 2): shell height 17.8–18.2 mm, shell breadth 35.4–37.2 mm, aperture height 10.4–11.0 mm, aperture width 16.6–17.5 mm, embryonic shell whorls 1.250–1.500, whorls 5.000–5.125, shell height/ breadth ratio 0.20–0.22.

General anatomy. Eversible head wart between ommatophore insertions absent. Tentacles and dorsum leaden-black. Sole and the remaining lower lateral side creamy white. Jaw arcuate; with 12 more or less projecting ribs (Fig. 4C).

Genitalia (Figs 4–6). A sheet of membrane (in Fig. 4A, the remnant membrane after dissection is arrowed), growing on oviducts and tightly connected with vas deferens, wrapping dart apparatus completely. Penis sheath very short; only restrictedly present near insertion on dart sac. Penis very short; moderately thick; externally simple. Penial retractor muscle inserting on penis-epiphallus transition. Epiphallus as thick as the most swollen part of penis; approximately two times longer than penis. Flagellum approximately three times longer than penis; tapering. Epiphallic papilla absent. Penis internally with four pilasters. Penial pilasters uniformly-spaced; thickest at two thirds of their entire length distally (Fig. 5B). Dart sac tiny (Fig. 5D). Dart in dart sac not observed (maybe lost or regenerating) in HBUMM06875 specimen-2. Accessory sac developed; large in size; interiorly with pilasters; transversally with developed sphincter muscles (Figs 5E, 6A, B). Mucous glands nine; extending distally to more than half of bursa copulatrix duct (longest in the subfamily Bradybaeninae); each thicker than penis; in volume larger than rest of genitalia (Fig. 4A); connected to each other by nerve fibres (Fig. 5C). Each mucous gland duct entering accessory sac through a separate pore; through papilla or not (Figs 5E, 6A, B). Vagina approximately as long as accessory sac; entering dart sac chamber. A ball-shaped proximal accessory sac (Figs 4A, 5A, D, E, 6A), about 1.5 mm in diameter, with opening leading to dart sac chamber near entrance of vagina. Gonad glands palm-shaped; with short peduncles (Fig. 4B). Measurement in HBUMM06875 specimen-2: DS–2.1 mm long; AS–4.1 mm long; MG–30.9 mm (average of 5 mucous glands); P–6.9 mm; Ep–10.3 mm; Fl–19.1; VD–22.0 mm; PR–3.2 mm; Va–3.2 mm; FO–4.9 mm; BC plus BCD–37.5 mm.

####### Distribution.

Extant distribution: Jiangxi (type locality: around Boyanghu; Lushan), Hubei (Ou-tang =Wudangshan Mts., Tong-san in Sei-zo), Fujian (Foochow= Fuzhou. Yen 1942), Zhejiang (Tianmushan Mts. This study). Pleistocene: Jiangsu (Chenkiang: Chiao-shan =Zhengjiang: Jiaoshan) (Fig. 1).

####### Ecology.

Perhaps this species is among the rarest bradybaenine species in China, although the extant distribution range is large and covers Jiangxi, Hubei, Fujian and Zhejiang. *Traumatophoratriscalpta* was known from higher altitudes of 1200–1500 m (Pilsbry 1890).

####### Taxonomic remarks.

In this study, the *Traumatophora* specimens from Zhejiang are identified as *T.triscalpta* based on the original description and comparison of the type material (Fig. 3). I follow Yen (1943) in treating *T.triscalpta* and *T.fraterminor* (originally described as *Helixtriscalptafraterminor* Gredler, 1884: 137. *T.triscalptafraterminor*Yen 1939: 126, pl. 13, fig. 8; Zilch 1974. *T.fraterminor*Yen 1943: 297) as two distinct species, although the only known difference between them is that the latter species is “smaller in diameter and higher in altitude, obtusely angulated at periphery” (Pilsbry 1890). However, considering the frequency of convergence events in the evolution of shell morphology in the helicoids, it is reasonable to treat these two taxa that show detectable differences in shell morphology as two species. *Traumatophorafraterminor* is distributed between Hwang-tchou (today’s Huangshi, Hubei Province) and Kiou-Kiang (today’s Jiujiang, Jiangxi Province) (Pilsbry, 1890) (Fig. 1). Report of a Pleistocene fossil from Zhenjiang, Jiangsu as *T.fraterminor* (sensu Yen 1943) is somewhat doubtful because the relevant specimen was a juvenile shell.

## Discussion

The anatomy of the terminal genitalia, especially the dart apparatus, varies greatly in the subfamily Bradybaeninae (Table 1) and is of considerable systematic significance. Most functions of these characteristic structures remain unclear, however,the function of some of these structures can be speculated to relate to the products of the dart apparatus, i.e., the love dart secreted in the dart chamber and the mucus secreted by the mucous glands, the function of which might be closely related to the reproductive success of the dart user as in the helicid snail *Cornuaspersum* (Lodi & Koene, 2016). *Traumatophora*, however, show an examples of the dart apparatus consisting of a tiny dart (proportionally the smallest in the Bradybaeninae) and disproportionately large mucous glands (proportionally the largest in the Bradybaeninae). Therefore, the genus *Traumatophora* provides a counter-example of the correlated evolution between stylophore and mucous glands (Koene and Schulenburg 2005: fig. 4A).

*Traumatophoratriscalpta* has a rounded proximal accessory sac with an opening directly leading to the dart sac chamber. A similar structure is also present in some *Stilpnodiscus* species (*S.moellendorffi* Wu, 2001: Wu 2001a, figs 4A, B, E, F; *S.entochilus* Möllendorff, 1899: Wu 2001a, figs 2A, B) and in the *Pseudiberus* spp. distributed in Shandong and Hebei (unpublished) (Table 1). The function of such sac-like structure is unknown, although it is predicted to be related to the storage of mucus secreted by the mucous glands. What is also fascinating is that in *Traumatophora* the occurrence of both the sphincter muscles in the accessory sac (Fig. 6A) and the developed nerve fibres connecting the mucous gland ducts (Fig. 5C, arrowed) suggests that during dart-shooting in mating, there might be an instant ejection of mucus by the animal.

**Table 1.** Comparison of characteristics of terminal genitalia among Chinese bradybaenine genera (Chang 1990; Wu 2001a, 2001b; Wu and Guo 2003; Schileyko 2004; Wu 2004; Wu and Guo 2006; Wu and Prozorova 2006; Wu and Asami 2017; Páll-Gergely et al. 2018; * this study). AS–accessory sac; DVM–membranous sac surrounding terminal genitalia; EpP–epiphallus papilla; Fl–flagellum; MG–number of mucous gland ducts; MGP–papilla distally leading to mucous glands on inner wall of accessory sac; PAS–proximal accessory sac, a blind sac on proximal dart sac and opening into dart sac chamber or not; PC--penial caecum; PLs--poly-layered structure in dart sac and/or accessory sac, produced by wavy and spongy connective tissue; PS–penis sheath.

	PS	EpP	PC	Fl	PLs	DVM	AS	PAS	MG	MGP
*Acusta* Martens, 1860	●	○	○	○	○	○	●	○	2	●
*Aegista* Albers, 1850	●	●	○	●	●	○	●	○	2	○
*Aegistohadra* Wu, 2004	○	○	●	●	○	○	●	?	2	○
*Armandiella* Ancey, 1883*	●	○	○	○	○	●	●	○	2	○
*Bradybaena* Beck, 1837	●	○	○	○	●	●	○	○	2	○
*Cathaica* Möllendorff, 1884	●	○	○	○	●	○	●	○	>2	○
*Coccoglypta* Pilsbry, 1895	○	?	○	○	?	○	●	○	2	?
*Dolicheulota* Pilsbry, 1901	●	●	○	●	?	○	●	○	>2	?
*Eueuhadra* Wu, 2004	○	○	●	●	○	○	●	○	>2	○
*Euhadra* Pilsbry, 1890	●	●	○	●	○	□	●	○	>2	□
*Karaftohelix* Pilsbry, 1927	●	○	○	○	○	●	●	○	>2	○
*Laeocathaica* Möllendorff, 1899	●	○	○	○	○	○	●	□	>2	○
*Landouria* Godwin-Austen, 1918	○	●	○	●	N/A	N/A	N/A	N/A	N/A	N/A
*Mastigeulota* Pilsbry, 1895	●	○	●	○	●	○	●	○	>2	○
*Mesasiata* Schileyko, 1978	●	○	○	○	○	○	●	○	>2	○
*Metodontia* Möllendorff, 1886	●	○	○	○	●	○	○	○	2	○
*Nesiohelix* Kuroda & Emura, 1943	○	○	□	●	○	○	●	○	>2	●
*Pancala* Kuroda & Habe, 1949	○	?	○	●	N/A	N/A	N/A	N/A	N/A	N/A
*Plectotropis* Martens, 1860	●	●	○	●	●	○	●	○	2	○
*Pliocathaica* Andreae, 1900	●	●	○	○	○	○	●	○	>2	○
*Ponsadenia* Schileyko, 1978	●	○	○	○	○	○	●	○	1	○
*Pseudaspasita* Möllendorff, 1901	●	?	○	●	●	○	●	○	2	○
*Pseudiberus* Ancey, 1887	●	○	○	○	○	○	○	●	>2	○
*Pseudobuliminus* Gredler, 1886	●	○	○	○	○	●	○	○	2	○
*Stilpnodiscus* Möllendorff, 1899	●	○	○	○	○	○	●	□	≥2	○
*Traumatophora* Ancey, 1887*	●	○	○	●	○	○	●	●	>2	●
*Trichobradybaena* Wu, 2003	●	○	●	○	○	●	●	○	2	○
*Trichocathaica* Gude, 1919	●	○	○	○	?	●	●	○	>2	?
*Yakuchloritis* Habe, 1955	○	?	○	●	N/A	N/A	N/A	N/A	N/A	N/A

● –present, ○ –absent, □ –present or not, ? –unknown, N/A –not applicable

## Supplementary Material

XML Treatment for Traumatophora

XML Treatment for Traumatophora triscalpta
